# Somatic Copy Number Alterations Associated with Japanese or Endometriosis in Ovarian Clear Cell Adenocarcinoma

**DOI:** 10.1371/journal.pone.0116977

**Published:** 2015-02-06

**Authors:** Aikou Okamoto, Jalid Sehouli, Nozomu Yanaihara, Yukihiro Hirata, Ioana Braicu, Byoung-Gie Kim, Satoshi Takakura, Misato Saito, Satoshi Yanagida, Masataka Takenaka, Noriko Yamaguchi, Asuka Morikawa, Hiroshi Tanabe, Kyosuke Yamada, Kosuke Yoshihara, Takayuki Enomoto, Hiroaki Itamochi, Junzo Kigawa, Noriomi Matsumura, Ikuo Konishi, Satoshi Aida, Yuko Aoki, Nobuya Ishii, Kazunori Ochiai, Tetsu Akiyama, Mitsuyoshi Urashima

**Affiliations:** 1 Department of Obstetrics and Gynecology, Jikei University School of Medicine, Tokyo, Japan; 2 Department of Obstetrics and Gynecology, Charité University Hospital, Berlin, Germany; 3 Department of Obstetrics and Gynecology, Samsung Medical Center, Sungkyunkwan University School of Medicine, Seoul, South Korea; 4 Department of Obstetrics and Gynecology, Niigata University Graduate School of Medical and Dental Sciences, Niigata, Japan; 5 Department of Obstetrics and Gynecology, Tottori University School of Medicine, Yonago, Japan; 6 Department of Obstetrics and Gynecology, Kyoto University Graduate School of Medicine, Kyoto, Japan; 7 Pharmaceutical Research Department 2, Research Division, Chugai Pharmaceutical Co., Ltd., Kamakura, Japan; 8 Laboratory of Molecular and Genetic Information, Institute of Molecular and Cellular Biosciences, The University of Tokyo, Tokyo, Japan; 9 Division of Molecular Epidemiology, Jikei University School of Medicine, Tokyo, Japan; The University of Hong Kong, CHINA

## Abstract

When compared with other epithelial ovarian cancers, the clinical characteristics of ovarian clear cell adenocarcinoma (CCC) include 1) a higher incidence among Japanese, 2) an association with endometriosis, 3) poor prognosis in advanced stages, and 4) a higher incidence of thrombosis as a complication. We used high resolution comparative genomic hybridization (CGH) to identify somatic copy number alterations (SCNAs) associated with each of these clinical characteristics of CCC. The Human Genome CGH 244A Oligo Microarray was used to examine 144 samples obtained from 120 Japanese, 15 Korean, and nine German patients with CCC. The entire 8q chromosome (minimum corrected p-value: *q* = 0.0001) and chromosome 20q13.2 including the *ZNF217* locus (*q* = 0.0078) were amplified significantly more in Japanese than in Korean or German samples. This copy number amplification of the *ZNF217* gene was confirmed by quantitative real-time polymerase chain reaction (Q-PCR). *ZNF217* RNA levels were also higher in Japanese tumor samples than in non-Japanese samples (*P* = 0.027). Moreover, endometriosis was associated with amplification of *EGFR* gene (*q* = 0.047), which was again confirmed by Q-PCR and correlated with *EGFR* RNA expression. However, no SCNAs were significantly associated with prognosis or thrombosis. These results indicated that there may be an association between CCC and *ZNF217* amplification among Japanese patients as well as between endometriosis and *EGFR* gene amplifications.

## Introduction

Epithelial ovarian cancer (EOC) is the 9^th^ most common cancer among women in the US, and the incidence of EOC in the US has remained relatively stable since 1992 [1]. In contrast, EOC is the 14^th^ most common cancer among women in Japan, and the incidence of EOC has been rising in Japan [2]. There are four major histological subgroups of EOC: serous, clear cell, endometrioid, and mucinous [3]. Among the EOC types, clear cell adenocarcinoma (CCC) is the third most common in North America and Europe with a reported prevalence of 1–12% [4–7]; notably, the prevalence of CCC in Japan is as high as 15–25% [8,9]. Furthermore, during the past two decades in Japan, CCC increased the most of the four subtypes, particularly in the 50+ age group [10]. Recent international collaborative studies confirm that endometriosis, which is a common gynecological disorder characterized by ectopic growth of endometrial glands and stroma [11], may increase the risk of CCC and of endometrial carcinomas of the ovaries [12]. The incidence of endometriosis among Asian women is reportedly nine times higher than that among Caucasian women [13]. Consistent with this data, the incidence of endometriosis has increased in Japan, possibly due to delayed marriage and decline in parity [10]. Thus, unique epidemiological links may exist among Japanese heritage, endometriosis, and CCC. Moreover, the majority of patients who are diagnosed with CCC at an early stage (I or II) have tumors that tend to be indolent, whereas patients in advanced stage (III or IV) usually have a poor prognosis [14,15]. Additionally, deep-vein thrombosis occurs in the perioperative period or during the course of postoperative chemotherapy in 14~27% of ovarian cancer patients [16–18]. Interestingly, patients with CCC have a 2.5-times greater risk of disease-related venous thromboembolism than those with other types of EOC despite adherence to prophylactic guidelines [19].

Subtypes of EOCs differ not only epidemiologically, but also molecularly. Mutations in *ARID1A* (*at-rich interaction domain-containing protein 1A*), a tumor-suppressor gene, and loss of the *ARID1A*-encoded protein, BAF250a, are evident in 46% of CCCs, 30% of endometrioid cancers, and many contiguous atypical endometriosis, but these changes are not observed in high-grade serous ovarian carcinoma [20,21]; these differences explain, at least in part, how these somatic mutations may trigger transformation of endometriosis into CCC or endometrioid carcinoma. Moreover, in CCC samples, DNA copy number gains are frequently observed at chromosome 20, which harbours a potential oncogene, *ZNF217* (*Zinc Finger Protein 217*), but this alteration is not observed in serous carcinoma samples [22]. Additionally, *phosphatidylinositol 3-kinase* (*PI3K*) mutations are relatively common in CCC samples, but *TP53* mutations are not [23]. Notably, a research network used integrated genome analyses and found that almost all high-grade serous ovarian cancer tumors harbor *TP53* mutations and that fewer but still statistically significant numbers harbor somatic mutations in *NF1*, *BRCA1*, *BRCA2*, *RB1*, *CDK12*, or some combination thereof [24]; these molecular characteristics are quite different from those of CCC. While these findings are important, they do not explain why CCC is more prevalent among Japanese, and they do not account for the associations between CCC and endometriosis or thrombosis. Therefore, we used oligonucleotide array techniques to identify genes associated with the prevalence of CCC among Japanese and correlations between certain clinical characteristics and CCC.

## Patients and Methods

### Patients and collaboration

This international collaborative study to detect important SCNAs in CCC was approved by the Ethics Committee for Biomedical Research of the Jikei Institutional Review Board, Jikei University School of Medicine, Tokyo, Japan. To conduct a post-hoc analysis, we asked Japanese (Jikei University School of Medicine, Tokyo, Niigata University Graduate School of Medical and Dental Sciences, Niigata, Tottori University School of Medicine, Yonago, Kyoto University Graduate School of Medicine, Kyoto), Korean (Samsung Medical Center, Sungkyunkwan University School of Medicine, Seoul), and German (Charité University Hospital, Berlin) collaborators to send surgical specimens and clinical information from patients with a pathological diagnosis of CCC.

### Specimens

Tumor specimens were surgically obtained from 144 patients; and each patient provided written informed consent. The histological diagnosis was confirimed by a central pathological review for all samples. Immunohistochemical analyses with CK7, CK20, estrogen receptor, progesterone receptor, WT1, and Hepatocyte nuclear factor (HNF)-1ß antibodies were also performed for confirmation of CCC. Entire operative specimens were collected as fresh-frozen material. Cryostat sections containing >80% cancer cells were microdissected [25]; DNA was extracted from these sections.

### Treatment

Patients underwent laparotomy for diagnosis, staging, and tumor debulking, and subsequently received first-line platinum-based chemotherapy. Tumor material for the study was excised at the time of primary surgery and before any chemotherapy. Surgical staging was determined in accordance with Fédération Internationale des Gynaecologistes et Obstetristes (FIGO) classification. Patients received Paclitaxel–Carboplatin treatment as a first-line chemotherapy. Among the cases, patients who did not recur for more than 1 year after six courses of chemotherapy were defined “Sensitive”. Patients who showed progressive disease during chemotherapy were classified as “Resistant”. All other patients were classified as “Intermediate”.

### Follow-up

Progression of disease was determined based on imaging and according to the Response Evaluation Criteria in Solid Tumors guidelines modified for ovarian cancer [26,27], or on physical examination. Progression-free survival (PFS) was defined as the interval between the end of primary treatment and the first indication of disease progression.

### Array comparative genomic hybridization (aCGH)

The Human Genome CGH 244A Oligo Microarray Kit (Agilent Technologies, Santa Clara, CA) was used. DNA digestion, labeling, and hybridization were performed according to the manufacturer's protocol. Briefly, each DNA sample from tumor (2 μg) or control (2 μg) tissue was digested with both Rsa I and Alu I; next, tumor and control DNA were labeled with Cy5-dUTP and Cy3-dUTP, respectively, and then mixed. Each DNA mixture was hybridized to human genome 244K microarrays. The slides were scanned with a DNA Microarray Scanner (Agilent Technologies). Data was extracted from scanned images using Feature Extraction software, version 10.7.3.1 (Agilent Technologies).

### Polymerase chain reaction

Quantitative polymerase chain reaction (Q-PCR) and a subset of 140 cases for which DNA was available were used to reexamine regions identified as exhibiting aberrant copy number alterations based on the CGH microarray data [28]. To normalize the copy number per cell, sequences from *ß-globin* and *WNT9A* genes were used as endogenous references. Q-PCR for each sample and each gene were performed in triplicate. DNA from three normal tissue samples that were randomly selected from resected specimen was used as the control.

Total RNA was isolated from fresh-frozen tumor samples using the Trizol reagent (Invitrogen, Carlsbad, CS, USA). cDNAs were synthesized from 3 µg of total RNA using the SuperScriptIII-Strand Synthesis System (Invitrogen Japan K.K., Tokyo, Japan). The cDNAs were used for real-time PCR (RT-PCR) analysis for *EGFR* and *ZNF217* expression. RT-PCR reactions were performed on an Applied Biosystems StepOnePlus platform using the TaqMan Fast Advanced Master mix.

### Mutational Analysis of Full-Length *EGFR*

DNA was extracted from tumor samples using the GentraPureGene kit (Qiagen, Tokyo, Japan). Exons 18, 19, and 21, which encode the tyrosine kinase domain, were amplified using primers as previous described [28]. PCR products were sequenced with the ABI PRISM BigDye Terminator Cycle Sequencing Ready Reaction kit and the ABI PRISM 3700 Genetic Analyzer (PE Applied Biosystems, Foster City, CA).

### Statistics

The Aberration Detection Method 2 quality-weighted interval score algorithm of Agilent Genomic Workbench Lite Edition 6.5.0.18 (Agilent Technologies), which identifies aberrant intervals in samples that have consistently high or low log ratios based on their statistical score, was used to assess penetrance of aberrant chromosomal regions across the genome; this analysis was followed with a filtering step to select regions with three or more adjacent probes and a minimum average log_2_ ratio ± 0.25. Gene Springs 11.5 (Gene Springs, Tokyo, Japan) was used to plot DNA copy number anomalies after excluding samples with poor quality. The genomic locations were retrieved from National Center for Biotechnology Information (NCBI) build 36 (hg 19). To compare data between two groups (e.g., patients with endometriosis vs. those without endometriosis) or three groups (e.g., response to treatment: sensitive vs. intermediate vs. resistant), an unpaired Mann-Whitney test and Kruskal-Wallis test were used, respectively; the Benjamini-Hochberg false-discovery rate was then used to correct the p-values. Probes with a corrected p-value (= q-value) less than 0.05 were considered statistically significant. The study data have been deposited in NCBIs Gene Expression Omnibus (http://www.ncbi.nlm.nhi.gov/geo/, series accession number GSE47443).

Analysis of variance (ANOVA), Kruskal-Wallis test, chi-square test, and a single-ordered logistic regression model were used to evaluate differences in patients' characteristics stratified by countries. The above statistical analyses were performed using STATA 13.1 (STATA Corp., College Station, TX). P<0.05 was considered statistically significant.

## Results

### Patients’ characteristics

Each of 144 patients from Japan, Korea, or Germany had their clinical data made available (Table 1). The median follow-up period was 24 months (range 2–191 months). The mean age was 53 years (range; 30–86 years old). The FIGO stages were as follows: Stage I, 85; stage II, 17; stage III 39; stage IV; 3. Of the 144 patients, 85 (71%) were considered chemotherapy sensitive, 12 (10%) were intermediate, and 22 (19%) were resistant. Of all patients, 43 (30%) relapsed and 29 (20%) died. Endometriosis was diagnosed in 68 (57%) of the Japanese patients, 6 (40%) of the Korean, and 2 (22%) of the German patients; these different prevalence of the endometriosis were statistically significant among compared populations (P = 0.041).

**Table 1:** Patient characteristics for each population. *^1^

Country (n)	Japanese (120)	Korean (15)	German (9)	Total (144)	P-value
Disease duration (months) median: IQR^*2^	26:15~50	10: 5~23	35: 16~59	24: 13~47	0.009^*3^
Age (years) mean (SD)	54 (11)	50 (8)	47 (11)	53 (11)	0.09^*4^
Stage, n I/II/III/IV	75/13/29/3	4/3/8/0	6/1/2/0	85/17/39/3	0.18^*5^
Sensitivity to chemotherapy, n					
Sensitive/intermediate/resistant	75/11/18	4/0/3	6/1/1	85/12/22	0.48^*5^
Progression, n (%)	36 (30)	3 (38)	4 (27)	43 (30)	0.86^*5^
Death, n (%)	26 (22)	2 (13)	1 (3)	29 (20)	0.64^*5^
Endometriosis, n (%)	68 (57)	6 (40)	2 (22)	73 (52)	0.041^*6^

*1: Japanese, Korean, German.

*2: IQR: interquartile range.

*3: P-value was evaluated with the Kruskal-Wallis equality-of-populations rank test.

*4: P-value was calculated with an analysis of variance and covariance.

*5: P-value was calculated with a chi-square test.

*6: P-value was evaluated with single ordered logistic regression model for populations (Japanese-Korean-German).

### Penetrance of aberration and differences among populations

**Figure 1:**
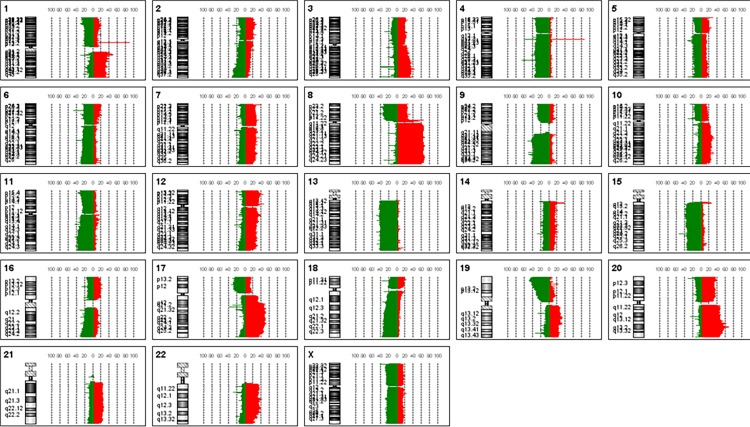
Frequency of SCNAs gain or loss for 22 autosomes and the X chromosome. Frequencies (%) of copy number gain (right side of central axis, red) and copy number loss (left side of central axis, green) across the human genome are shown.

Frequencies of SCNA gains and losses are shown in Fig. 1. Across the entire genome, the most prevalent SCNAs were either very short (focal level), almost exactly the length of a chromosome arm (arm level), or the length of a whole chromosome (chromosome level). Chromosome amplification was most frequently observed (>60%) at 8q, and chromosome loss was most frequently observed (>40%) at 9q, at 13q and 17q.

**Figure 2:**
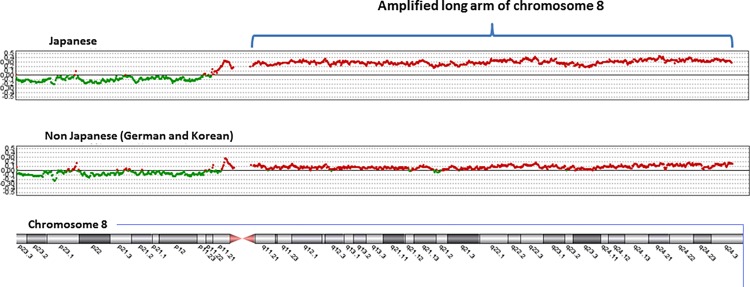
Comparison between Japanese and Koreans or Germans with regard to SCNAs on all of chromosome 8. Horizontal center line indicates the normal copy number without loss or gain.

Many loci differed significantly between Japanese and non-Japanese (Korean and German) samples (S1 Table). At the level of a chromosome arm, all of chromosome 8q was significantly more amplified in 63% of Japanese samples, but only in 21% of Korean (P = 0.003) and 7% of German samples (P = 0.005) (Fig. 2).

**Figure 3:**
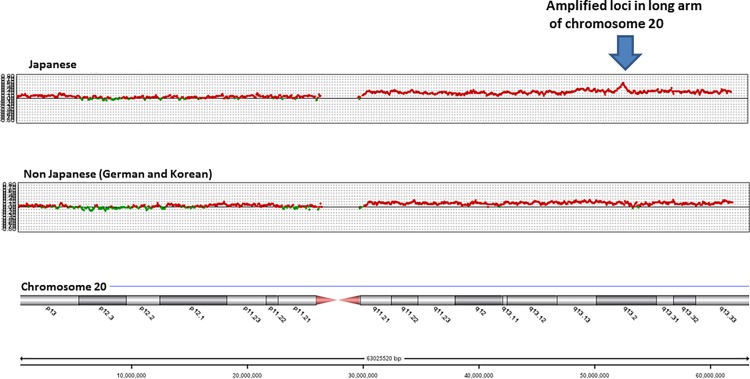
Comparison between Japanese and Koreans or Germans with regard to SCNAs on all of chromosome 20.

**Figure 4:**
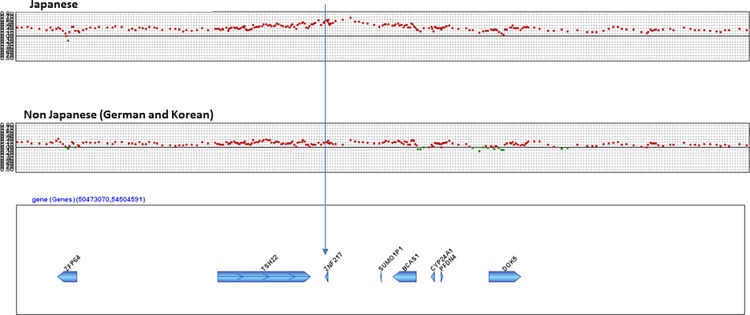
Comparison between Japanese and Koreans or Germans with regard to SCNAs on chromosome 20q13.2.

**Figure 5:**
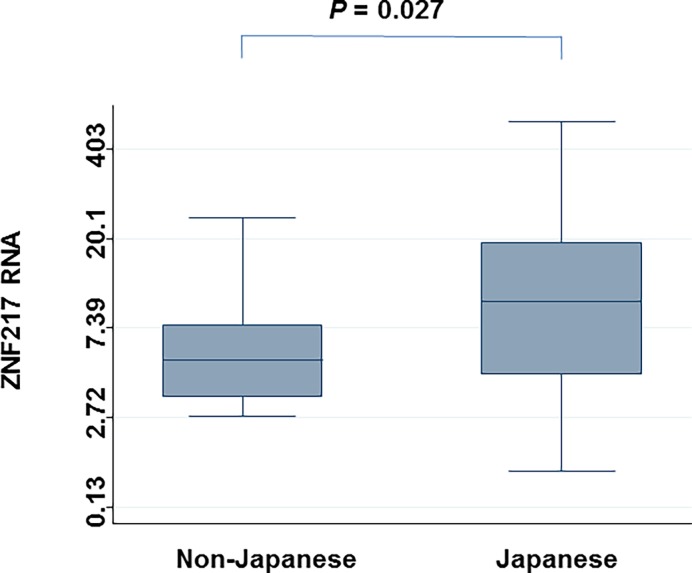
Comparison of *ZNF217* RNA expression between CCC samples from Japanese and non-Japanese patients. P-value was calculated with the Mann-Whitney test.

Additionally, there was remarkable amplification of loci in chromosome 20q13.2 (including *ZNF217*) in Japanese samples, but not in non-Japanese samples (Figs. 3 and 4) (minimum q = 0.0078). When individual CGH data was surveyed, *ZNF217* copy number was altered (amplified) in 62% of Japanese samples, which was significantly more than that for Korean (7%, P = 0.001), or German (25%, P = 0.040) samples. To confirm amplification of *ZNF217* gene copy number originally identified via CGH, Q-PCR was used for validation and showed high agreement (81%) and high kappa values (63%). Moreover, *ZNF217* RNA levels were significantly higher in Japanese samples than in non-Japanese samples (P = 0.027) (Fig. 5).

### Endometriosis and somatic copy number alterations

**Figure 6:**
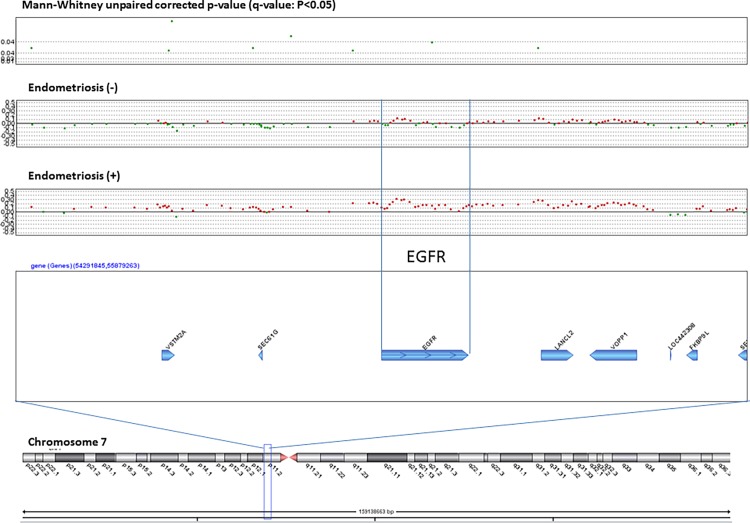
SCNAs associated with endometriosis. Significant q-values (<0.05) (y-axis) (upper), SCNAs without endometriosis and with endometriosis (middle) and gene map are plotted around a region that includes the EGFR gene on chromosome 7.

**Figure 7:**
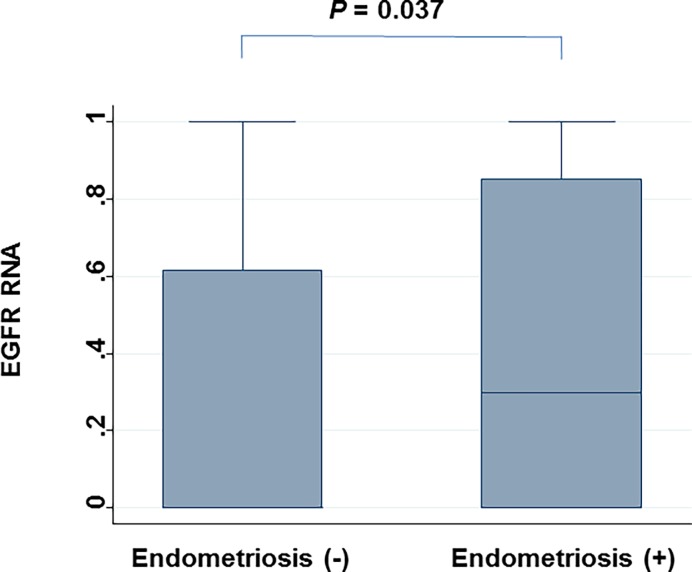
Comparison of *EGFR* RNA expression between CCC samples from patients with endometriosis and those without endometriosis. P-value was calculated with the Mann-Whitney test.

We analyzed the q-values of SCNAs associated with clinical characteristics of CCC. No SCNAs were found to be significantly associated with relapse, death, FIGO stage, sensitivity to chemotherapy, or thrombosis. In contrast, patients with endometriosis and those without differed significantly with regard to 135 SCNAs (S2 Table), and the locus that includes *EGFR* gene amplification was most prominent (Fig. 6). Based on CGH data and validating Q-PCR data from individual sample and high agreement (86%) and high kappa values (70%), the prevalence of endometriosis was significantly higher in patients with an amplified *EGFR* locus (85%) than in those without *EGFR* amplification (35%) (*P*<0.0001). Moreover, based on RT-PCR analysis, *EGFR* RNA levels were significantly higher in samples from patients with endometriosis than from those without it (P = 0.037) (Fig. 7). In contrast to SCNAs, no pathological mutations of *EGFR* were observed (data not shown).

## Discussion

In this study, we used the Human Genome CGH 244A Oligo Microarray to investigate CCC. Chromosome aberrations were observed throughout the genome. However, amplification was the most frequently observed at 8q, and chromosome loss was the most frequently observed at 9q, 13q, and 17q that was reported by Tan et al [29]. The amplification of 8q and loss of 13q observed were consistent with findings from a previous study, although the previous study was conducted in the setting of a broader histological presentation, and was not restricting to CCC samples [30].

We attempted to identify SCNAs that were significantly associated with each of four epidemiological features of CCC: 1) a higher incidence in the Japanese population, 2) an association with endometriosis, 3) poor prognosis in advanced stages, 4) a high incidence of thrombosis as complication. First, we tried to find Japanese-specific SCNAs in CCC. There were a large number of significant SCNAs in Japanese samples that were distinct from those in Korean or German samples. However, accumulation of significant regions in whole chromosome 8q (arm level) was noticed. Moreover, the chromosome 20q13.2 region, which includes *ZNF217* (focal level), tended to be amplified specifically in Japanese tumor samples, but not in the Korean or German samples; this findings was confirmed by Q-PCR. Moreover, *ZNF217* RNA levels were significantly higher in Japanese samples than in non-Japanese samples. *ZNF217* was initially found to be amplified in breast cancer and considered to work as an oncogene [31]. Huang et al, suggested that *ZNF217* may promote neoplastic transformation by increasing cell survival during telomeric crisis, and may promote later stages of malignancy by increasing cell survival during chemotherapy [32]. Later, *ZNF217* amplification was reported not in ovarian serous carcinoma, but in CCC [22]. Recently, Huang et al. showed that loss of *ARID1A* expression was usually coincident with PI3K-Akt pathway activation and/or *ZNF217* amplification; they suggested that these abnormalities may be related to the development of CCC [33]. Rahman et al. demonstrated that *ZNF217* amplification correlated with shorter PFS [34], but we did not replicate this finding.

Secondly, our findings indicated that amplified *EGFR* SCNAs were more frequently observed in patients with endometriosis than in those without it. The activation of EGFR, via over-expression of the receptor, the ligands, or structural alteration, has shown that EGFR signalling plays a critical role in cell proliferation, apoptosis, angiogenesis, and metastasis [35]. Based on immunohistochemistry findings, EGFR protein is expressed in endometriosis specimens [36]. Association of *EGFR* gene amplification in patients with both CCC and endometriosis is a novel research finding, although no associations between *EGFR* gene polymorphisms with endometriosis [37] and with endometrioid carcinoma [38] were reported previously. A site located in CDKN2BAS on chromosome 9p21 that encodes the cyclin-dependent kinase inhibitor 2B antisense RNA was reported as a susceptibility locus for endometriosis [39]. This study was conducted as a genome-wide association study, in contrast our study was based on CGH using cohort of patients with CCC. Amplification of *EGFR* SCNA was reported in colorectal cancer [40], esophageal cancer [41], glioblastomas [42], lung cancer [43], salivary gland cancer [44], and osteosarcoma [45]; taken together, these findings indicated that SCNA of *EGFR* is relatively common in cancer tissue. However, we could not determine whether amplification of *EGFR* triggered the progression from endometriosis to carcinoma or whether this amplification resulted from carcinogenesis because design of this study was cross sectional.

There are several limitations to this study. First, this study was performed as an international collaboration among multiple institutions. Sampling and fresh-freezing procedures may have differed among institutions or countries. Second, only a small number of countries were compared including Japan, Germany, and Korea. Due to this small sample size, statistical power was limited and could not be used to identify important SCNAs. Additionally, there were only 24 non-Japanese samples, which make comparisons difficult. Also, there are likely to be significant differences between Korean and German samples. Third, we were able to used Q-PCR and RT-PCR to confirm that the *ZNF217* and *EGFR* genes were amplified and their RNAs were overexpressed, but we could not examine protein expression because paraffin-embedded samples were available for an insufficient number of patients.

In conclusions, amplification of whole chromosomes 8q and 20q13.2 including the *ZNF217* gene were more prevalent in Japanese CCC samples than in samples from other populations. *ZNF217* RNA levels were also higher in CCC samples obtained from Japanese patients than those from non-Japanese patients. Moreover, amplification and RNA over-expression of *EGFR* gene were significantly higher in CCC tissue of patients with endometriosis than from those without it. In this study, we did not find significant associations between specific SCNAs and FIGO stage, sensitivity to chemotherapy, disease progression, death, or thrombosis as a complication.

## Supporting Information

S1 Table Data of genes different between Japanese and non-Japanese. (XLSX)

S2 Table Data of genes different between patients with and without endometriosis. (XLSX)
